# RoboCoV Cleaner: An Indoor Autonomous UV-C Disinfection Robot with Advanced Dual-Safety Systems

**DOI:** 10.3390/s24030974

**Published:** 2024-02-02

**Authors:** Dragoș-Vasile Bratu, Maria-Alexandra Zolya, Sorin-Aurel Moraru

**Affiliations:** Department of Automatics and Information Technology, Transilvania University of Brasov, 500036 Brașov, Romania; alexandra.zolya@unitbv.ro (M.-A.Z.); smoraru@unitbv.ro (S.-A.M.)

**Keywords:** UV-C disinfection robotics, disinfection solutions, dual-safety systems, sensor-fusion AI system for human detection

## Abstract

In the face of today’s ever-evolving global health landscape and ambient assisted living (AAL), marked by the persistent emergence of novel viruses and diseases that impact vulnerable categories and individual safety, the need for innovative disinfection solutions has surged to unprecedented levels. In pursuit of advancing the field of autonomous UV-C disinfection robotics, we conducted two comprehensive state-of-the-art analyses: the first one in the literature and the second one in existing commercial disinfection robots to identify current challenges. Of all of the challenges, we consider the most outstanding ones to be safeguarding humans and animals and understanding the surroundings while operating the disinfection process challenges that we will address in this article. While UV-C lamps have demonstrated their effectiveness in sterilizing air and surfaces, the field of autonomous UV-C disinfection robotics represents a critical domain that requires advancement, particularly in safeguarding the wellbeing of humans and animals during operation. Operating UV-C disinfection robots in close proximity to humans or animals introduces inherent risks, and existing disinfection robots often fall short in incorporating advanced safety systems. In response to these challenges, we propose the RoboCoV Cleaner—an indoor autonomous UV-C disinfection robot equipped with an advanced dual and redundant safety system. This novel approach incorporates multiple passive infrared (PIR) sensors and AI object detection on a 360-degree camera. Under our test, the dual-redundant system reached more than 90% when detecting humans with high accuracy using the AI system 99% up to 30 m away in a university hallway (different light conditions) combined with the PIR system (with lower accuracy). The PIR system was proved to be a redundant system for uninterrupted operation during communication challenges, ensuring continuous sensor information collection with a swift response time of 50 ms (image processing within 200 ms). It empowers the robot to detect and react to human presence, even under challenging conditions, such as when individuals wear masks, in complete darkness, under UV light, or in environments with blurred visual conditions. In our test, the detection system performed outstandingly well with up to 99% detection rate of humans. Beyond safety features, the RoboCoV Cleaner can identify objects in its surroundings. This capability empowers the robot to discern objects affected by UV-C light, enabling it to apply specialized rules for targeted disinfection. The proposed system exhibits a wide range of capabilities beyond its core purpose of disinfection, making it suitable for healthcare facilities, universities, conference venues, and hospitals. Its implementation has the ability to improve significantly human safety and protect people. By showcasing the RoboCoV Cleaner’s safety-first approach and adaptability, we aim to set a new benchmark for UV-C disinfection robots, promoting clean and secure environments while protecting vulnerable people, even in challenging scenarios.

## 1. Introduction

In the context of a pandemic, as witnessed during the rapid spread of the SARS-CoV-2 virus, the urgent demand for quick and effective disinfection methods, such as UV lamps deployed in indoor environments, became quite apparent. Different research [1,2] has underscored the efficacy of mask-wearing in curbing the transmission of certain diseases, but it remains imperative to ensure the thorough disinfection of surfaces. Consequently, eliminating pathogens from the air carries substantial advantages for all.

Contaminated surfaces significantly raise the risk of disease transmission by allowing pathogens to spread through contact or air. Proper disinfection procedures reduce the likelihood of disease transmission. A safe life is ensured by effective and frequent disinfection of one’s surroundings. As there are numerous hard-to-reach spots or inaccessible regions, such as upper corner walls and ceilings or narrow shelves, a typical cleaning approach utilizing cleaning solutions by humans alone will not be able to minimize the quantity of these microorganisms. In this direction, UV-C light is one of the most effective disinfection solutions available [3,4]. According to [5], the SARS-CoV-2 virus resists on surfaces for a period of 6 h to 9 days, and it is destroyed by ultraviolet light, making it effective for other viruses, bacteria, or even certain types of mold harmful to humans [5]. This type of light can assist hospitals and similar facilities in their constant battle against the persistence of bacteria and viruses in patient rooms, preventing the potential for new infections. The C band of UV light, spanning 200–280 nm, effectively eliminates microorganisms, including bacteria, viruses, and fungi, like molds, but its wavelength can also affect human eyes and skin. Autonomous robotic systems have gained significant attention across industries for their performance and advantages, with the benefit of safeguarding human health by reducing risk exposure. In this context, the proposal to use a robot to disinfect the rooms using lamps with UV-C light has many advantages. First of all, it is autonomous, with very little human intervention necessary. Additionally, it does not involve the use of more chemicals, which could harm humans and equipment even after use, with some improvement for human detection during disinfection.

**Figure 1:**
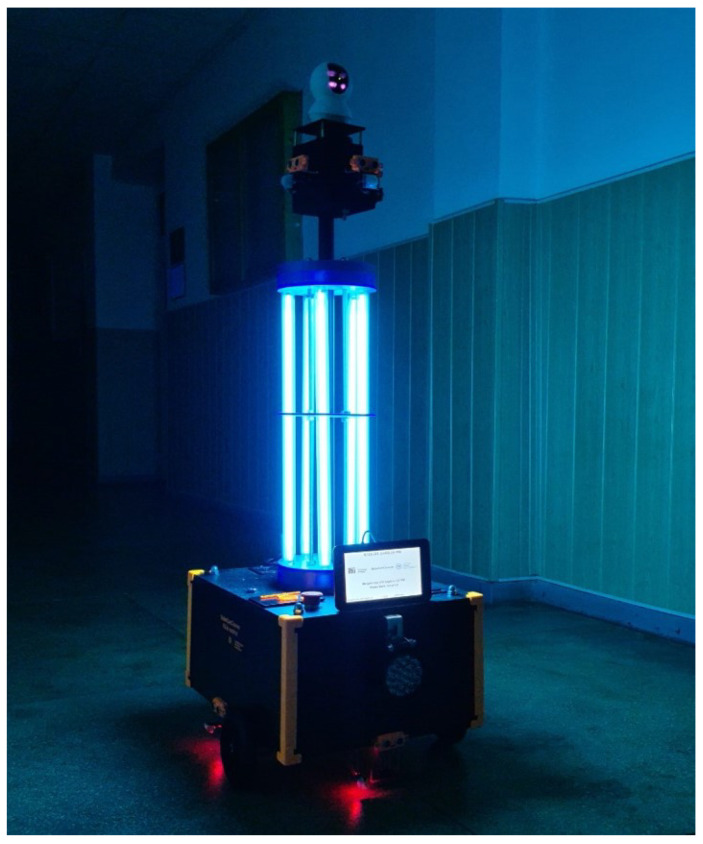
RoboCoV Cleaner robot disinfecting a Transilvania University of Brașov hallway.

This paper presents the RoboCoV Cleaner robot, shown in Figure 1. It is an autonomous robot equipped with five UV-C lamps and two redundant systems for detecting human presence. Additionally, it has multiple sensors and specialized disinfection capabilities and can identify objects even under challenging conditions.

## 2. Overview of Existing UV-C Disinfection Robot Systems, Their Designs, and Effectiveness

Technological advancements in recent years have facilitated the development of sophisticated disinfection systems, notably characterized by the emergence of UV-C disinfection robots. These robotic devices use the germicidal characteristics of UV-C light to provide a significant level of pathogen eradication in diverse configurations. As we explore the domain of UV-C disinfection robots, it is important to evaluate their diverse designs and assess their efficacy rigorously. This chapter aims to provide insight into different systems, focusing on their distinct objectives, inherent benefits, possible drawbacks, and essential functions.

Table 1 provides a comprehensive depiction of the estimated price range, derived from several sources, demonstrating that these robotic devices are linked to substantial costs, often exceeding an average of USD 50,000. A common feature seen in these systems is the use of UV-C lamps, a well-established method that has shown efficacy in mitigating the prevalence of illnesses.

The robot systems in question provide a variety of mobility options, which range from movable bases [7,9,10,11,13,15] that may move in any direction on the floor, focusing the vertically fixed UV-C lights wherever needed, to stationary platforms that use vertically or horizontally oriented UV-C sources, as seen in [8,12,16] systems.

Significantly, certain systems exhibit additional functionalities that distinguish them from others. An example of this may be seen in the Helios robot [10], which utilizes a parabolic reflector to enhance its disinfection effectiveness and concentrate the UV-C light. In addition, several robot versions include quartz UV-C lamps and spray systems to enhance their disinfection capabilities. Nevertheless, when we delve into analysis, a multidimensional picture becomes apparent. The Honeywell approach [12], for example, has a stationary platform that requires manual manipulation by human operators, often in confined environments such as airplane cabins or buses. In this context, the use of protective equipment becomes a necessary need, serving to protect operators from possible risks associated with exposure to UV-C radiation. Within the domain of UV-C-equipped robots, the pursuit of human presence detection has resulted in diverse levels of integration. While several systems may claim to possess just one detection mechanism, our analysis uncovers an intriguing divergence.

An apparent gap in current commercial disinfection systems is the absence of advanced human detection capabilities. Many existing robots lack sophisticated systems in this regard, often relying on a single system or none at all.

When exploring the intricacies of advanced features found in current commercial disinfection robot systems, our focus naturally shifts towards the findings outlined in Table 2. The table serves as an informative collection, providing a thorough summary of other characteristics that engage our interest. Within this context, it is essential to delve into the features that surpass traditional limits so that we can find potential challenges that can be solved.

One of the first enhancements is the ability to provide live video feedback, which allows end-users to engage in real-time surveillance and maintain constant monitoring of the disinfection process. This dynamic aspect moves beyond the scope of simple mechanization, enabling operators to determine the operational wellbeing and effectiveness of the system at any given point in time. The capacity to see the operational environment and supervise the process of disinfection appears to be an important point that enhances trust and transparency in operations.

The emergence of autonomous capabilities is also a significant milestone in the development of disinfecting robots, enabling them to execute complex disinfection protocols with less human involvement. This has the potential to decrease operational bottlenecks, enhance efficiency, and highlight the technological advancements that challenge traditional limitations. As can be seen, there is a trend to create robots that work autonomously, but there is still room for improvement. Many of these advanced systems lack the capability of real-time object recognition from the live feed, which, in our opinion, is an important aspect. Furthermore, the lack of adequate capabilities for simple upgradability and integration with developing new sensing technologies undermines the prospects of these robots. The limitation to a particular sensor generation can potentially restrict future improvements, necessitating a thorough assessment of the system’s durability and capacity to adapt, and, mainly, it is offering just software updates. When examining the time aspect of disinfection, an overall trend emerges, indicating an average disinfection duration of around 2–2.5 h. Moreover, some commercial systems extend their scope to include other disciplines through user-friendly apps, providing remote access and facilitating administration and software updates [7].

Following a comprehensive analysis of the current state of scientific literature pertaining to disinfecting robots, a notable and captivating trend emerges, as seen in Table 3.

The G-Robot [19] demonstrates that there is a noticeable trend towards the integration of robotic arms with various degrees of freedom in addition to the prevalent categorizations of mobile [18,20,23,24] and static bases [22] for these robots. Within the context of movable and static base systems, a common structure arises, the same as the commercial ones, distinguished by the presence of vertically oriented UV-C lights in [18,20,22], etc.

The uniform orientation is consistently implemented across different systems, each adopting unique configurations (some horizontally, some vertically). It is important to note that many of these systems exhibit a deficiency concerning a complete method for detecting human presence. The lack of this crucial safety element creates a vulnerability whereby any miscommunications among controllers (central unit and actuator unit) overseeing distinct components could compromise the overall operation while disinfecting. Moreover, the omission of redundancy, a fundamental principle in the field of safety engineering, necessitates a thorough reassessment of system resilience and the associated risks. To cover this aspect, some systems tend to use alternative UV-C wavelengths [19], which are considered inherently safer (far-UVC lamps). However innovative, the use of these lamps has not been demonstrated to have the same efficacy as UV-C lamps over known pathogens.

Examining extra characteristics in the context of advanced disinfection robot systems within the academic literature, presented in Table 4, unveils a landscape that has similarities to, but also possesses different characteristics from, their commercial counterparts. In this particular approach, several aspects formerly confined to commerce systems are now considered essential principles for academic investigation. Significantly, the concepts of real-time live feeds and enhanced autonomy, which have become more important in business settings, remain topics of interest. These specified features are fundamental elements in academic investigation, driving research efforts toward realizing their utmost capabilities.

Nonetheless, a noteworthy remark highlights the endeavor to explore undiscovered territories, placing special attention on the advanced system to detect humans or animals, recognize objects in the live video feed, understand current scenes, and generate reports. There is still room for improvement on aspects such as abstracting away hardware implementation from the fundamental logic of the robot. The concept of strategic decoupling illustrates a significant transformation in the design field, as it liberates the design landscape from previous limitations and encourages the exploration of modular possibilities, leaving room to improve on the next iterations.

### 2.1. Efficiency of UV Light over Known Pathogens

The primary concept behind the usage of UV-C lamps is their ability to induce damage to the DNA of bacteria and viruses, resulting in the sanitization of targeted areas. Nucleic acids are damaged, exposed to the germicidal wavelengths of UV light, their RNA and DNA are altered, and they can no longer reproduce properly. National Emerging Infectious Diseases Laboratories (NEIDL) (NEIDL is a state-of-the-art research facility comprising significant isolation laboratories located in Boston, MA, USA) studied the efficiency of the UV-C lights on some materials that contain the SARS-CoV-2 virus. It was discovered that a dose of 5 mJ/cm^2^ applied for 6 s resulted in a reduction of 99% of this virus, and a dose of 22 mJ/cm^2^ would result in a reduction of 99.99% in 25 s. UV-C lamps destroy viruses, bacteria, fungi, etc., but they also harm humans. For this reason, the robot was equipped with two systems for detecting people, which will be discussed in more detail in the next sections (Section 3.2).

The part of the electromagnetic spectrum between 200 and 400 nm is known as the ultraviolet zone. The rays emitted by the sources in this zone are invisible. The UV zone is divided into three sections: UV-A (320–400 nm), UV-B (280–320 nm), and UV-C (200–280 nm). Because UV-C rays have a relatively high energy level, direct contact with the eyes and skin should be avoided. Historically, three types of UV lamps have been used for germicide purposes: lower-pressure mercury lamps (LPM), pulsed xenon flash lamps (PXF), and far-UVC (known as excimer lamps). LPM lamps are remarkably similar to conventional fluorescent lamps in shape and form and are monochromatic as they produce UV light at 253.7 nm (∼254 nm). PXF lamps emit germicidal ultraviolet light in flashes lasting a few milliseconds. Pulsed xenon produces a wide UVC wavelength spectrum ranging from 200–315 nm and can combine the germicidal effects of UV-C lighting with the thermal disintegration of cell walls due to the intensity and speed of photonic delivery. The third type of lamp, the far-UVC lamp, is less harmful than the LPM and PXF. Far-UVC light uses 207–222 nm wavelengths, and it has been shown to be effective [25] in destroying some microorganisms as conventional sterilizers that use UV lamps, but analyses to date demonstrate that these wavelengths do not cause human health issues associated with direct exposure to conventional germicidal UV light.

#### 2.1.1. Dose Needed to Inactivate an Organism

The dose needed can be thought of as the levels of UV light that an organism receives. It is measured in millijoules per square centimeter (mJ/cm^2^).

(1) $$Dosage\;\left({\mathrm{mJ}/\mathrm{cm}}^{2}\right)=Irradiance\;\left({\mathrm{mW}/\mathrm{cm}}^{2}\right)\times Duration\;\left(s\right)$$

Irradiance is the amount of radiant power reaching a surface per unit area. The photon flux is measured in watts or milliwatts per square centimeter (mW/cm^2^). Irradiance values vary according to lamp output power, the direction of its reflector system, and the distance to the surface that needs to be disinfected. In summary, this depends not only on the lamp quality but also on the robot implementation, such as where the lamps are positioned, how far the robot is positioned from a surface of interest, and so on.

**Figure 2:**
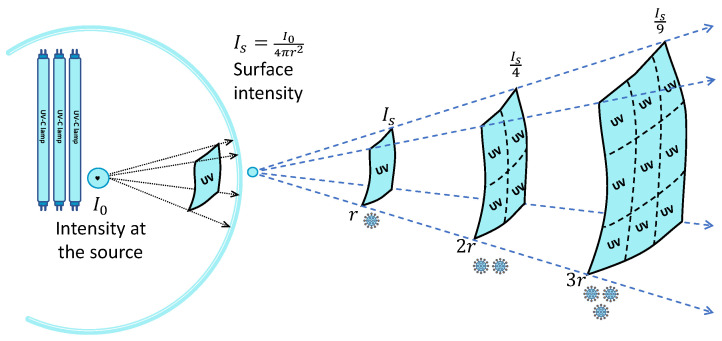
The inverse square law from a source point. $I_{s}$ is the intensity at the surface, $I_{0}$ is the source intensity in watts, and *r* the radius of the sphere, adapted after the source [26].

The distance to the surface of interest plays a crucial role as UV radiation follows the inverse square law, as seen in Figure 2. To conclude, the irradiance power multiplied by the exposure time gives the dosage, or how much the robot needs to keep the lights on, as seen in the dosage Formula (1).

#### 2.1.2. UV-C Light Effective for Disinfecting Coronavirus

According to German Medicine, Science, Hygiene, and Infection Control [27], coronaviruses do not differ structurally from other viruses. This means that the SARS-CoV-2 virus and potential future mutations will most likely be highly UV-sensitive. Standard UV-C disinfection procedures will inactivate the new SARS-CoV-2 virus without changing the technology of this type of disinfection light. The upper limit determined for the 1 log-reduction dose (90% reduction) is approximately 10.6 mJ/cm^2^ and the true value is probably only 3.7 mJ/cm^2^ (median) [27]. Similar studies confirm the above [28,29,30,31]. National Emerging Infectious Diseases Laboratories (NEIDL) concluded that a dose of 22 mJ/cm^2^ would result in a reduction of 99.99% in 25 s [32].

### 2.2. Challenges or Areas of Improvement in Existing Solutions

In light of worldwide health emergencies and AAL, there has been a significant emphasis on utilizing technological advancements to combat the spread of infectious illnesses. An example of an innovative technology is the disinfection robot, as presented in the above section, both in the commercial and academic worlds, which has been specifically engineered to cleanse areas using techniques such as UV-C radiation. As with any groundbreaking technology, disinfection robots present a distinct set of challenges that necessitate consideration and resolution, some of them highlighted in Section 2. According to our analysis, the following enumeration highlights the challenges linked to various disinfection robotic systems:

1. Safety concerns, particularly when considering human exposure. UV-C light has been known to cause injury to the skin and eyes of humans. It is of utmost importance to ensure that these robots do not subject anyone to UV-C radiation;
2. The cost of UV-C disinfection robots remains quite substantial, hence restricting their availability to certain businesses and people from certain categories to innovate;
3. Battery life and operation time of the disinfection robots systems, which usually need frequent recharging that will correspondingly result in a decrease in efficiency;
4. Efficacy of the required UV-C dosage for thorough cleaning of surfaces can present difficulties. The presence of shadows or inaccessible places may result in an unequal distribution. The efficacy of UV-C can be impacted by various environmental factors, including humidity levels, ambient temperature, etc.;
5. Material degradation of some materials, such as plastics, with extended exposure to UV-C light or other chemicals. Moreover, when UV-C lamps are used, they can break down oxygen molecules in the air, leading to the production of ozone;
6. Navigation, control, and accessibility. Disinfection robots often rely on human control for precise operation, particularly when navigating intricate environments. This becomes evident in multilevel buildings where maneuvering stairs presents a notable challenge or where new objects appear in the scene;
7. Versatility, scalability, and integration with other systems. Disinfection robots grapple with issues of versatility and scalability, particularly when adapting to diverse environments and tasks. Furthermore, their integration with existing systems poses significant challenges, particularly within complex infrastructural settings and when they are designed for one purpose;
8. Maintenance and upgrading parts. Maintenance tasks, such as recalibrating sensors or replacing worn-out wheels, used filters, or burned-out UV-C lamps, are recurrent necessities for disinfection robots. Furthermore, in the context of technological advancements, it becomes imperative to upgrade systems with newer components, such as a more efficient UV light module or updated navigation software. However, the integration of these components may sometimes pose compatibility challenges;
9. Usability and on-boarding for the users that often encounter challenges with the interfaces of these robots, which tend to be nonintuitive, hence requiring extensive training. Additionally, the initial configuration process in new environments may be time-consuming. Moreover, there exists a noticeable reluctance among prospective users, which is mostly based on a deficiency of trust and confidence in the effectiveness and reliability of these systems;
10. Continuous monitoring and reporting to the functioning of these robots in order to maintain effective disinfection. Nevertheless, some systems exhibit a deficiency in their capacity to provide instantaneous feedback (of the system and also live video) or deliver it in a form that is readily comprehensible to the end-users. Furthermore, the lack of comprehensive reporting channels may impede the evaluation of sustained effectiveness and the detection of areas that need improvement.

### 2.3. Current Proposal

Recently, there have been notable developments in the field of robotics and artificial intelligence which have facilitated the emergence of new solutions to meet the needs of people with specific requirements [33,34,35] in a context where the aging population is creating an increasing demand for health services.

The proposed system presents a strategic approach to effectively mitigate some of the existing disinfection robot solutions challenges presented in Section 2.2. This system addresses the challenges of human safety, efficacy, battery life, and material degradation by offering an object recognition system, navigation while disinfecting, reports, and versatility for new technologies.

The framework improves operational safety by strengthening human detection via the use of redundancy systems located in both device units. The system excels in human and object recognition across the UV, visible, and infrared spectrum, as presented in Section 3.2, augmenting its efficacy in diverse environments and scenarios.

At its essence, this innovation introduces the concept of decoupling logical part of hardware implementation details, enabling an efficient method of adaptation and leaving room for improvements in the subsequent iterations of robots by quickly adding new sensors and actuators. This system provides real-time insights through live system updates and remote control. The framework accommodates specialized disinfection programs, tailoring its capabilities to varying contexts. The proposal maintains affordability by utilizing easily accessible components (open-source components), ensuring widespread accessibility.

## 3. Design and Functionality of the System

### 3.1. RoboCoV Cleaner Robot Design

**Figure 3:**
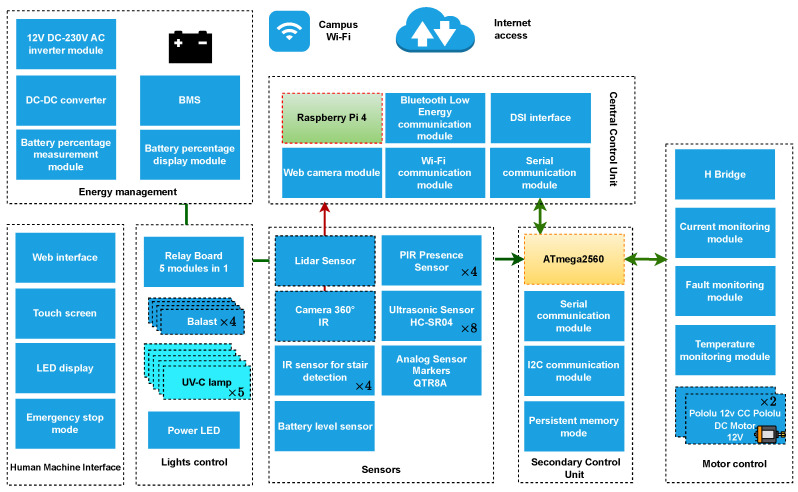
Block diagram illustrating the integrated system, showcasing the interconnections and relationships among its constituent components.

The robot is a complex mechatronic system, requiring expertise in mechanics, electronics, automatics, and software development, including image processing and real-time operating systems. The block diagram is presented in Figure 3.

This type of robot brings numerous advantages to its users, the most important of which will be described in the following paragraphs. With the benefit of UV light, the robot is able to quickly disinfect any room, such as classrooms, laboratories, bathrooms, or even hallways (Figure 1), being effective not only at short distances but also at longer distances. The UV light-powered robot can last up to 8000 h without the need for chemicals or consumables, requiring only a 12 V battery charge upon discharge. Unlike other methods that require human intervention, this autonomous disinfection process uses markers (as detailed in Section 3.1.2) and is not dependent on chemicals or consumables. Another advantage is that it can also be controlled remotely using a web-based application developed in Flask, so the only thing needed is a connection to the internet and a browser. Through it, it can be turned on or off or send commands to move inside and outside the room. Through the attached camera, the user can monitor the area where the robot operates. It can also operate at night (in the complete dark), as the camera has an IR filter, but it can also work in autonomous mode using the markers described in Section 3.1.2. The attached display can be used for many functions, from displaying the battery percentage and the remaining time until the device is charged to displaying various messages, such as emergency messages. As was previously mentioned, it is essential that it can detect humans. That being said, a double detection system was used: in addition to the PIR sensor, the robot is able to detect the presence of people in the room using the inbuilt camera image processing and machine learning, as described in Section 3.2. The camera will also be utilized to read QR codes, which will be placed at the entrance of each room. Each code will contain the necessary information for the robot about the room, such as the possibility of sanitizing the room (there are rooms where disinfection is not desired, for example, the guard room or any other room), as described in Section 3.1.2. After all the rooms have been sanitized, the robot enters standby mode.

During the implementation of the project, it was necessary to design certain components for building and supporting the robot. The UV lamp holder was made using the design shown in Figure 5 in the Autodesk Inventor Professional 2019 v4 tool. Other components, such as colored washers, an ultrasonic sensor holder, a LiDAR case holder, etc., were also designed. The 3D design was chosen because there are no components already made that fully meet our requirements, and the low cost of this approach was also an important decision factor.

**Figure 4:**
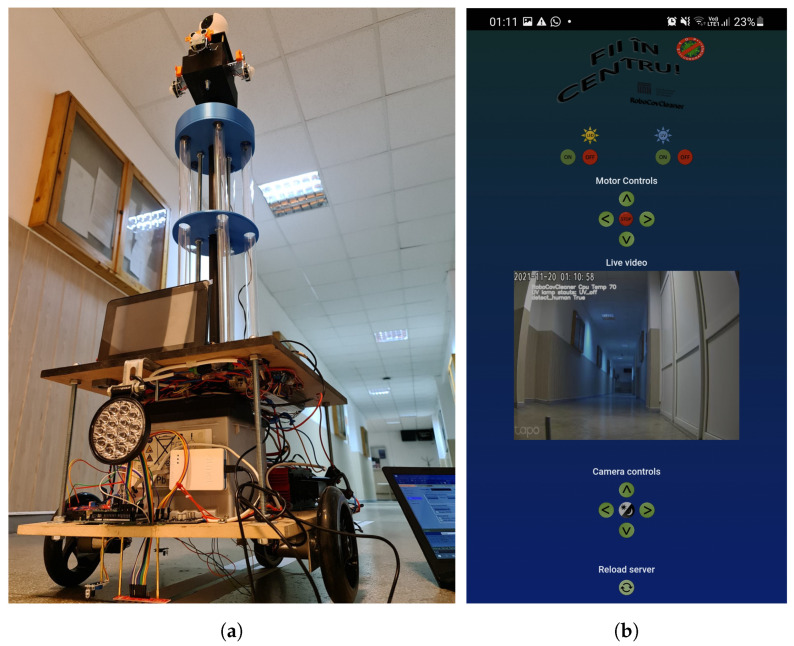
The RoboCoV Cleaner robot, fully assembled before the final aesthetic touch, captured from a lower perspective as it navigates the corridors of the University (**a**) and its web interface (**b**).

As can be seen in Figure 4a, the base of the robot was made of MDF plywood, to which two L-shaped supports were firmly attached to adequately support the DC motors. In the later stages, in order to obtain a solid and sturdy foundation, M10-sized nuts were used, giving a height of about 30 cm. It is worth mentioning that the height selection was not random but was determined by the requirements needed to install the 12 volt battery. Thus, an additional MDF plywood plate was added on top of this battery, and the whole structure was enclosed to provide adequate protection.

**Figure 5:**
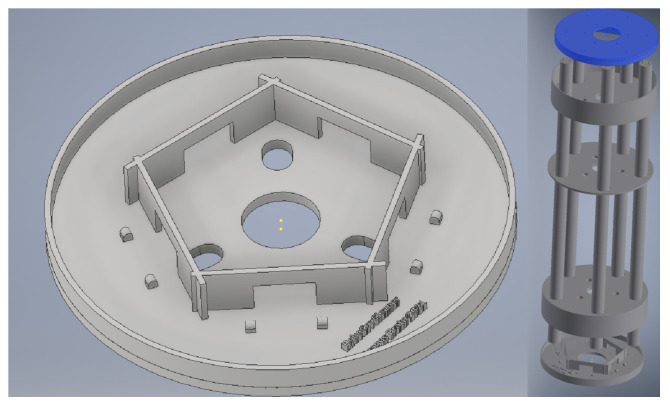
Design and evaluation of 3D-printed robotic elements; screenshot from Autodesk Inventor 2019.

In the next steps, the ultraviolet light lamps were fixed on supports made at the 3D printer (Figure 5), with an aluminum pipe in the center attached to the robot’s base. The aim was to keep the greatest weight at the base of the robot to keep the center of gravity as low as possible, which helped to avoid potential tipping of the device.

#### 3.1.1. Sensors and Actuators Used in the System

An important aspect of this robot was to have an electrical system that was as simple as possible, to reduce costs. At the same time, the user’s safety was a top priority, as 220 V is needed for the UV-C lamps and ballasts. The robot has five 18 watt Philips UV-C lamps with five ballasts, a power inverter for providing 220 V to the lamps, a 12 volt car battery (which can last for up to 4 h depending on the mode—see Equation (4)), and different sensors and actuators, as seen in Table 5.

This robot has a multitude of sensors that are necessary for detecting humans, obstacles, stairs, walls, or the battery level. The presence (PIR) sensor is used to detect the presence of a human or an animal. This is an important aspect, as the UV-C light’s interaction with human or animal skin or eyes can cause serious injuries. The infrared (IR) sensors are used to detect stairs or gaps under the robot, and in case something like this happens, the disinfection process is stopped. For measuring the space that needs to be disinfected, resulting in how much time the robot should have UV lamps turned on, an RPLiDAR A1 sensor is used to measure the distances in the room. In short, one of the purposes of the LiDAR sensor is to map the room in which the robot is located. Depending on the mapping result, the time it takes for the robot to disinfect a room can be calculated, taking into account its surface (this is only available in manual mode, and other modes can be implemented with ease). For detecting obstacles, a total of eight ultrasonic sensors are used, four in the lower part and four in the upper part. This design was preferred in order to protect the UV lamps in the event that some desks are encountered. A line follower approach was embraced to enforce location constraints for the robot’s movement, employing an IR sensor for line detection. Based on different markers on the floor, the robot can take different actions that can stop the lamps (when sensitive equipment is nearby) or even the disinfection if it is not needed. The battery detection sensor is a simple sensor made from a voltage divider to display the battery not only on the robot’s platform but also on the online interface.

The experimental setup includes a 12 V lead–acid battery with a capacity of 41 Ah, six 18 W UV-C lamps (powered by an inverter), a Raspberry Pi module (2.5 W), a Tapo camera (5 W), an Arduino Mega (0.5 W), and sensor and actuators with a total power of 118 W, according to the formula found in Equation (2).

(2) TotalPower(W)=P.L.+P.C.U.+P.S.+P.A.I.

where P.L. is power of the UV-C lamps, P.C.U. represents the average power of the control units, P.S. is estimated power of the sensors + P.A.I. is estimated power of the actuators and inverter.

TotalPower(W)=108W+7W+0.5W+2.5W=118W

Assuming the inverter is 100% efficient (which is an ideal case—efficiency may be lower), the current drawn from the battery can be calculated using Equation (3):

(3) $$\mathrm{Current}\;\left(A\right)=\frac{\mathrm{Total}\;\mathrm{Power}\;\left(W\right)}{\mathrm{Voltage}\;\left(V\right)}$$

which converts to

$$\mathrm{Current}\;\left(A\right)=\frac{118\;W}{12\;V}\approx 9.83\;A.$$

Maximum operating time in hours while using full disinfection can be computed with the following Equation (4):

(4) $$\mathrm{Time}\;\left(h\right)=\frac{\mathrm{Battery}\;\mathrm{Capacity}\;\left(\mathrm{Ah}\right)}{\mathrm{Current}\;\left(A\right)}$$

resulting in:

$$\mathrm{Time}\;\left(h\right)=\frac{41\;\mathrm{Ah}}{9.83\;A}\approx 4.17\;h$$

Using the low-energy mode where one of the UV-C lamps is powered on, we can reach up to 17.8 operating estimated hours.

$$\mathrm{Time}\;\left(h\right)=\frac{41\;\mathrm{Ah}}{2.3\;A}\approx 17.82\;h$$

The robot has two powerful 12 V brushed DC motors with a metal gearbox, which are controlled by an Atmel Atmega2560 (Atmel Corporation, based in San Jose, California, USA) microcontroller using a high-power H-Bridge driver. The robot uses different relays to control the UV-C lamps. For the low-power mode of the robot, the UV-C lamps can be turned off with the lowest setting of one lamp on. It is also equipped with a powerful 5 W LED used to see in hallways far apart. This was proven to raise the efficiency of human detection (first of all, the human will see it and be aware), with the only limitation being battery autonomy becoming lower (up to 1 h decrease). The actuators and sensors are represented in the block diagram of Figure 3.

#### 3.1.2. Control System

The robot has two operating modes: an autonomous one, in which it follows certain markers to know what it should do, and a manual mode, when the robot is desired to be controlled from a safe place by using a web app, as illustrated in Figure 4b.

The interface can be accessed through the browser directly from a mobile phone or a computer. After accessing the application, the soundings can be seen in real time with the attached camera. The user is able to move the view of the live camera within almost 360 degrees to analyze the area where the robot is and also to move the robot in the direction needed. Another necessary aspect of the remote application is that the user has the possibility to turn on or off the UV lamps or the power LED that is assembled on the front of the robot, which gives high control of the system. Manual mode overrides autonomous mode. This is used in emergencies or if the user wants to access out-of-scope areas. Therefore, with all of the above, the user has full access to control the robot to start the disinfection in the area desired and to safely turn it off.

**Figure 6:**
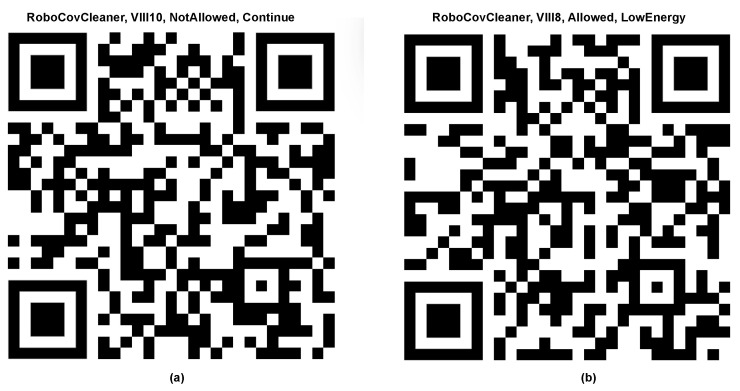
Examples of QR code which dictates what rules are to be followed: not allowed to enter a room (**a**), allowed to enter a room and use low-energy mode (**b**).

**Figure 7:**
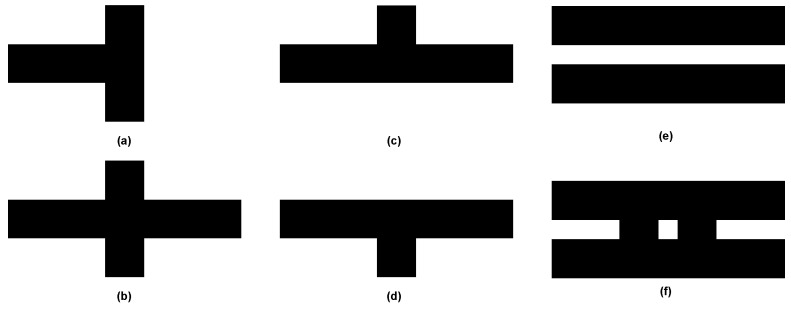
Examples of the decision floor markers and possible use-cases: read existing QR code and the turn of 180° (**a**), intersection check left and right QR codes and decide the next steps (**b**), read left QR code and decide the next steps (**c**), read right QR code and decide the next steps (**d**), start UV-C lamps here (**e**), stop UV-C lamps here and start scanning with the 360°camera for QR codes—protected area (**f**).

Furthermore, the enhancement of the robot’s capabilities is achieved by implementing specific disinfection programs. The mentioned systems, in addition to QR codes (Figure 6), use strategically positioned markers on the floor (Figure 7) in order to provide designated pathways for the robot. The proposed approach presents a viable substitute for the use of simultaneous localization and mapping (SLAM) methods, allowing meticulous regulation of the robot’s locomotion inside specified zones. Significantly, the diversity of the system enables the creation of exclusion zones, enhancing the efficacy and safety of disinfection procedures.

#### 3.1.3. Software Implementation

The software design is also elaborate because it is a complex system with many sensors, actuators, and programs. The software’s complexity actually benefits because the robot’s logic is easy to change or adapt depending on the case, thanks to scripts written in Python 3.7, a programming language close to the human language. The software complexity is due to using two different systems with different architectures and technologies. One is a microcontroller (Atmel Atmega2560) running an embedded real-time operating system called FreeRTOS, and the other is the Raspberry Pi 4 (manufactured by the Raspberry Pi Foundation, located in Cambridge, United Kingdom) using the Linux operating system (Debian 10.5—Buster). Combining two distinct platforms adds an extra layer of complexity to software development and integration. Raspberry Pi was programmed with the main role being the control board that will control the MCU board by communicating through the serial interface. This method is similar to the leader–follower model (The leader–follower model is a new adaptation of the established master–slave concept) where the leader (or driver) is the Raspberry Pi board.

The secondary sensor and actuator Uunit (USSA) is a software and hardware module that was specifically designed to abstract the hardware layer. It is also intended to manage sensor data processing, control any actuators in the system, and facilitate communication with the control central unit (UCC). The system was built using an Atmel Atmega2560 microcontroller (Atmel Atmega2560 is a 16 bit RISC architecture microcontroller developed by Microchip Technology (formerly Atmel Corporation)) with an operating frequency of 16 MHz, which was selected based on its specifications and the extensive pin availability it offers (100 pins).

**Figure 8:**
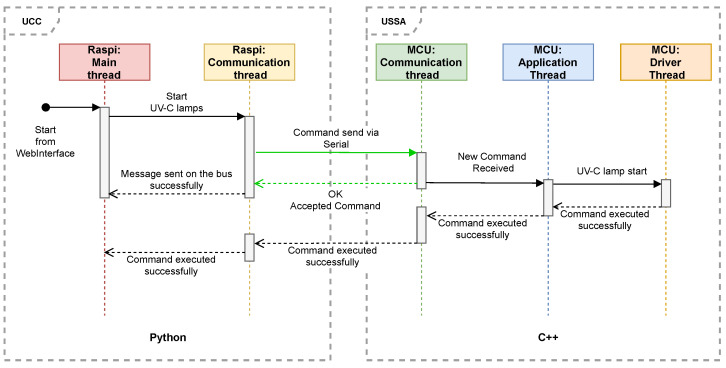
Sequence diagram between the two logical units UCC and USSA for sending a command and the related execution threads.

In order to make the most efficient use of resources and optimize system responsiveness, a real-time operating system, FreeRTOS, offering multitasking capabilities, was chosen. For software development, Atmel Studio 7 was used as a development environment together with a debugging board specifically designed for this microcontroller that facilitates the identification and efficient resolution of problems encountered during the development process, thus increasing the efficiency of debugging activities during software development using the Atmel Ice debugger. The software architecture running on USSA incorporates several distinct threads of execution for better segregation of tasks. The execution threads for both UCC and USSA are also shown in Figure 8.

### 3.2. Advanced System Implementation for Human Detection Using Machine Learning Techniques

Given the sensitivities arising from human and animal presence surrounding the robot, particular emphasis is placed on the detection system for both entities. The 360-degree camera provides input imagery for an intelligent system, utilizing the YOLO (You Only Look Once) algorithm [36,37], intermittently alongside the HAAR algorithm [38,39,40]. This combined approach aims to identify individuals within the robot’s visual field.

Upon successful human detection, immediate dimming of the lights ensues. Following a set interval, the camera reassesses for ongoing human presence, thereby ensuring a safe resumption of disinfection if necessary. The YOLO algorithm [36,37] excels in high-performance object detection, analyzing entire images to predict boundaries and class probabilities efficiently. Its single-stage process combines object localization and classification, yielding real-time data processing.

In contrast, the HAAR technique [38,39,40] uses rectangular filters to detect patterns, making it suitable for tasks like facial recognition. HAAR gained prominence in computer vision before deep-learning-based methods like YOLO. These algorithms are interchangeable and contingent on processing power for specific object detection.

## 4. Results

### 4.1. Advanced System Results for Human Detection

**Figure 9:**
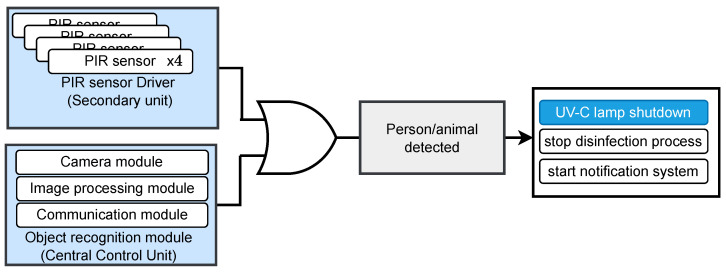
Logical block diagram of a dual-redundant human/animal detection system integrated into both logical controllers.

**Figure 10:**
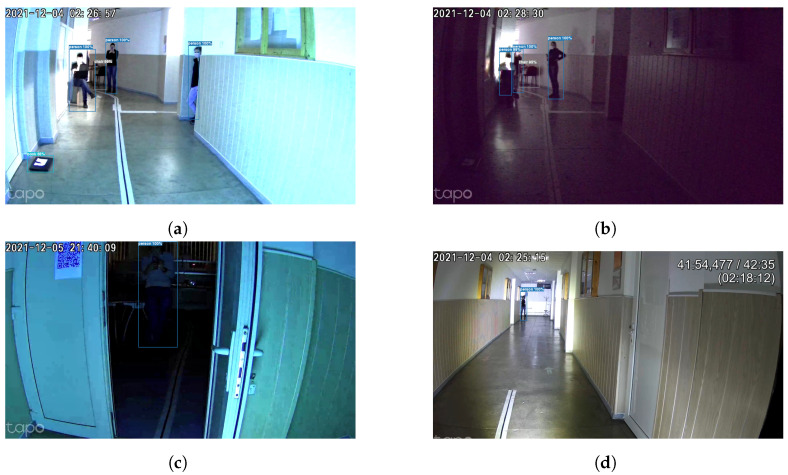
Human detection system under different lighting conditions using visible light filters. (**a**) Human detection with the UV produced by the robot as a light source. (**b**) Human detection in low-light conditions without using any source from the robot. (**c**) Human detection of an adjacent, dark room and UV light produced by the robot. (**d**) Human detection using the high-power LED at a distance of about 30 m.

**Figure 11:**
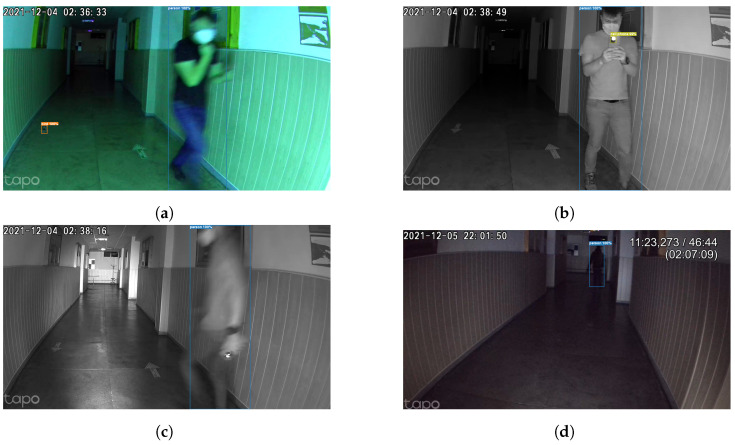
The human detection system in different lighting conditions using the infrared light filter, blurred pictures, or even masked people. (**a**) Human detection, UV light, moving subject with FFP2 protective mask. (**b**) Human detection IR light, moving subject with FFP2 protective mask. (**c**) Human detection IR light, moving subject with FFP2 protective mask. (**d**) Human detection poor normal lighting, at a distance of about 30 m.

As can be observed in Figure 9, Figure 10 and Figure 11, the use of dual-system techniques for human detection has resulted in a significant improvement in the effectiveness of human detection. The experimental findings clearly demonstrate the device’s efficacy over various illumination conditions, including UV, infrared, and low-light situations, and even blurry images with humans with FFP2 protective masks. Remarkably, the technology demonstrated the capacity to identify individuals at distances of up to 30 m inside the university hallway with up to 99%, as can be seen in Figure 10. Despite the higher effectiveness of object detection using the camera as input in comparison to PIR sensors, it is crucial to emphasize that these sensors, even if they have lower performance (smaller distances 5–30 m with a lower percentage of 70–80% of detecting humans, with different conditions), are useful when the central unit meets difficulties in picture transmission and processing the images. Including this supplementary safety measure guarantees uninterrupted operational efficiency, even when there are interruptions in communication between the primary and secondary units. This measure serves to protect the ongoing collection of sensor information. The PIR sensor has a response time of 50 ms, while the subsequent image processing and reaction occur within 200 ms, ensuring sufficient time for effective system operation.

### 4.2. Disinfection Program Using Specific Markers and Rules

**Figure 12:**
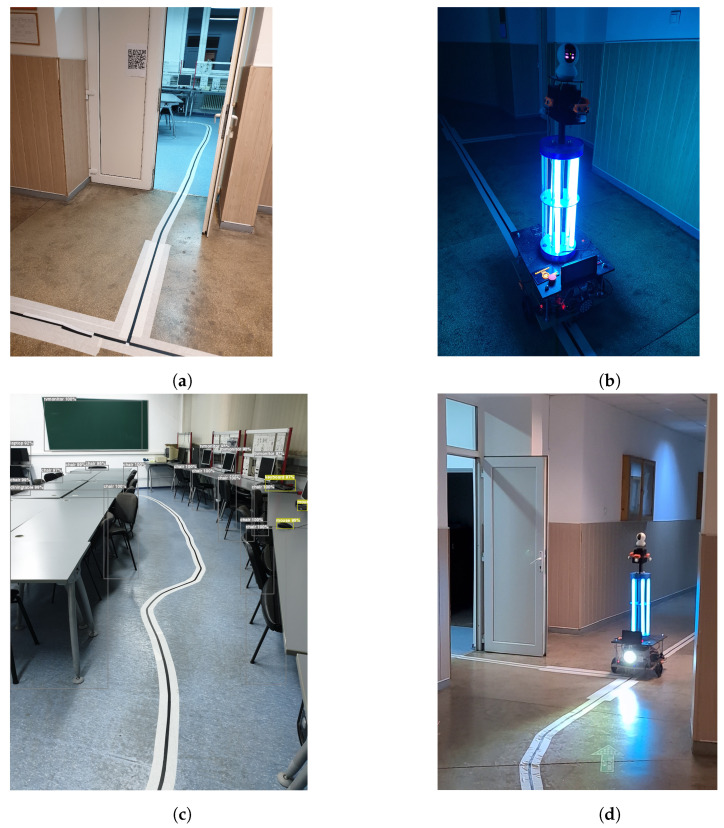
Autonomous mode of disinfection using markers. (**a**) Markers were placed on the floor, and a QR code was placed on the door with the disinfection rules to follow. (**b**) RoboCoV Cleaner using markers to disinfect the hallway of Transilvania University of Brașov. (**c**) Markers placed on the floor can have different paths, and the object detection algorithm can be used to understand the soundings. (**d**) RoboCoV Cleaner using markers placed on the floor and QR code placed on the door to make decisions at intersections.

The system’s control effectiveness is enhanced by the integration of a 360-degree camera that is connected to the control unit. Significant information may be collected by scanning QR codes placed along hallways and doors (as presented in Figure 12).

The QR codes serve as an effective way of transmitting necessary data, including the probability of infection and specific disinfection protocols. The aforementioned makes a flexible resource that can be used to enhance efficiency and ensure accuracy in many contexts. It is worth noting that changes to marker interpretation rules may be conveniently implemented on the control unit using the Python programming language. In addition, these markers might serve to enhance navigation efficiency, allowing the robot to effectively return to its original starting position. The system’s flexibility, accuracy, and multifunctionality are emphasized by integrating these many characteristics.

### 4.3. Object and Scene Recognition Insights

**Figure 13:**
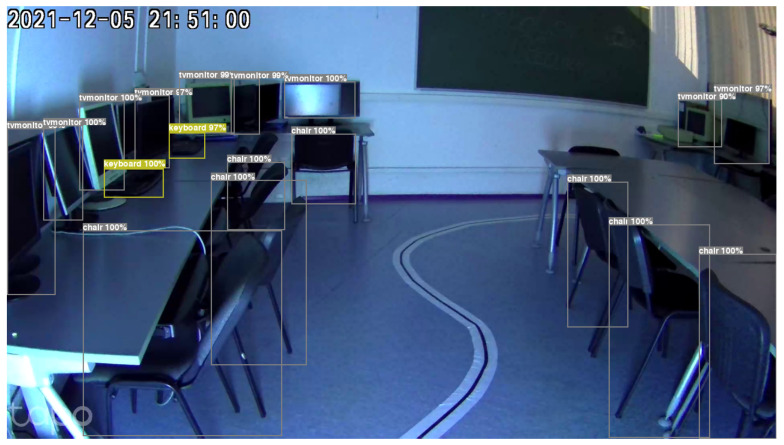
Object detection in a room while cleaning with UV light.

In addition, the system has capabilities beyond human detection, as seen in Figure 13. The detection system showcases the ability to identify and acknowledge various entities, including animals and objects, situated inside enclosed environments. Moreover, the system has the capability to provide extensive statistical data, enhancing its performance with an additional layer of analytical understanding. This can be used to better understand how many objects, what type, and if the UV light will affect them in the long term.

During the experimental analysis, the optimal disinfection distance and time for a robot were determined, which should be 2–2.5 m, and the time should be 10 s for hitting a certain surface for a prolonged disinfection time and retaining a great battery life for the robot. Having the robot in autonomous mode improves battery life, but having a determined path means efficiency in terms of area to clean.

## 5. Conclusions

This article presents a complex and complete indoor autonomous UV-C disinfection robot with a novel advanced dual and redundant safety system alongside existing challenges and current gaps in state-of-the-art systems in both commercial and academic domains. This novel approach enhances the robot’s ability to detect and react to human presence, an important feature even under challenging conditions, such as when individuals wear masks, in complete darkness, under UV light, or in environments with blurred visual conditions. Under our test, the dual-redundant system reached more than 90% accuracy when detecting humans using the camera live feed and artificial intelligence techniques (up to 30 m away in a university hallway, and achieving up to 99% accuracy Figure 10) combined with the PIR system. While camera-based detection outperforms PIR sensors, the latter, despite the lower performance, prove to be important for uninterrupted operation during communication challenges, ensuring continuous sensor information collection with a swift response time of 50 ms and subsequent image processing within 200 ms.

Through a comprehensive examination of disinfection robot navigation, the system proposed stands out with its innovative use of markers on the floor and QR codes on doors. This unique approach, distinct from LiDAR systems, underscores the contribution to the evolution of different cost-effective autonomous navigation systems used in disinfection systems.

This system addresses the challenges of human safety, efficacy, battery life, and material degradation by offering object recognition, human detection system, navigation while disinfecting, reports, and versatility for new technologies.

Its main core consists of UV-C light, which has been proven effective in inactivating molds, bacteria, and even the SARS-CoV-2 virus [41,42]. The robot has two operating modes: an autonomous one, in which it follows certain makers to know what it should do, and a manual mode, when the control of the robot is desired to be made from a safe place by using a web application. Due to its versatility, it can be upgraded with new components, and the main logic can be changed easily due to the abstraction of the hardware components.

### Future Improvements

Despite the RoboCov Cleaner robot system’s complexity and agility, and the fact that the robot reached its initial goals, we have to mention the limitations imposed by its design, which can be improved in the next generation of robots by learning from the previous generation. Security becomes a major issue, especially when considering how the robot might be controlled from outside over the internet. Implementing strong authentication and encryption mechanisms becomes essential in order to protect against possible vulnerabilities. Another aspect concerns privacy and who will have access to its live video feed and the data acquired in the reports. The human detection system can be greatly improved using another type of sensor, e.g., ultrasonic [43] fused with existing systems.

Additionally, the UV-C disinfection process ozone production is a challenge for the environment and humans. A solution may be to include carbon filters in the system, lowering ozone emissions and bringing technology in line with sustainable standards.

Another practical restriction is added by the necessity that the robot finds and docks at a charging station on its own, which calls for the creation of smooth docking systems. Additionally, increasing the control unit’s computing capacity and object identification algorithms might improve the system’s capability to successfully traverse challenging settings.

The potential future range of applications for this technology covers a wide range of areas, including providing personalized care and support and promoting social engagement, education, and environmental monitoring for specific patients. These applications may be as follows:

- Personal assistants: people with specific needs can benefit from using the robot’s artificial intelligence capabilities to receive personalized assistance. For example, the robot has the potential to provide reminders for medication schedules, engage in basic conversations to alleviate feelings of loneliness, or provide prompts for daily activities such as performing certain physical exercises;
- Monitoring: this robot is able to perform environmental monitoring by using its sensors and cameras. This allows the robot to observe and measure a range of environmental factors, including temperature, humidity, and air quality, as well as identify potential hazards such as gas leaks or fire outbreaks (using other sensors [44]). Moreover, the existence of a system that eliminates contact between people is essential for communicable, contagious diseases like COVID-19;
- Delivery and logistics: this robot may have the ability to move certain packages and, at the same time, be equipped with compartments or racks to facilitate the transport of items within a given unit. This application has potential value in healthcare facilities such as hospitals or nursing homes, where the safe and efficient transport of medical supplies, medicines, or food trays is of great importance;
- Social inclusion: this can be facilitated through the use of robots for the elderly, disabled, or those living in care homes. These robots can be specially designed to participate in interactive games, storytelling sessions, or even to lead group exercises. This facilitates interpersonal engagement and cognitive stimulation with the potential to enhance holistic wellbeing;
- Education: the robot can be programmed to be deployed in educational environments, thus helping certain perhaps disadvantaged groups participate in certain interactive training sessions. This could prove particularly advantageous for students with learning difficulties or those who need extra assistance;
- Virtual locomotion: for people with movement problems, such as paralysis or other problems, it is essential that they do not lose contact with the outside world. Thus, if the robot is equipped with a motion and video system, the patient can move virtually to certain physical locations. There are already such robots;
- Autonomous surveillance device: the robot can enhance security measures by integrating sophisticated camera systems and facial recognition technology. This allows it to effectively monitor access points, identify unauthorized persons, and conduct regular surveillance patrols in specified areas;
- Cost reduction: in special needs care centers, cost reduction is an important aspect. The automation of certain tasks can significantly contribute to achieving different goals.

The aforementioned concepts serve as examples to highlight the multifunctionality of the robot, which extends beyond its primary purpose of disinfection. Through the use of artificial intelligence and low-energy devices, the robot has the ability to be adapted and adjusted to meet a wide range of requirements in different situations. As a result, it can provide significant help and support to people with special needs as well as deal with the support of specific categories of people, thus proving to be a versatile and highly beneficial system.

## Figures and Tables

**Table 1:** State of the art in disinfecting robot commercial systems, evaluation of disinfection system, and human detection system.

Name	Price Range ^1^	Disinfection System	Human Det. System
Xenex Lightstrike [6,7]	USD 100,000–USD 125,000	Mobile base with 1×PXF-UV-C	4×PIR Sensor
Tru-D Smart UV-C [8]	USD 100,000–USD 125,000	Static base with 12×Double LPM UV-C	NO
UVD Robot [9]	USD 60,000–USD 100,000	Mobile base with 8×LMP UV-C	2×RGB-D Camera
Helios [10]	USD 90,000–USD 100,000	Mobile base with 5×LMP UV-C, parabolic reflector	Motion sensors not on the robot
BKS-UVRobot-200 [11]	N/A ^2^	Mobile base with 10×Quartz Lamp UV-C	NO
Honeywell Cabin System [12]	USD 10/single use	Static base with multiple size LMP UV-C	NO
Keenon M2 Robot [13,14]	USD 41,000–USD 50,000	Mobile base with 4×LPM UV-C and spray(15L)	NO
SIFROBOT-6.59 [15]	USD 20,000–USD 25,000	Mobile base with 6×LPM UV-C	NO
ZENZOE [16]	USD 90,000–USD 100,000	Static/Mobile base with 4×LPM UV-C	NO
FARYUAN FYB-K3 [17]	N/A ^2^	Mobile base with 4×Quartz Lamp, 2×LED UV-C and spray	NO

^1^ The price range provided is a rough approximation derived from several online sources at the time of 2021–2022. To obtain an accurate pricing, it is often necessary to submit a formal request, since the final cost may fluctuate depending on factors such as date, quantity, and specific features. ^2^ N/A stands for not applicable, as the price was not found.

**Table 2:** State of the art in disinfecting robot commercial systems’ extra features.

Name	Live F. ^1^	OR ^2^	Ctrl. ^3^	Aut. ^4^	Report	Upgr. ^5^	Op. Time ^6^
Xenex Lightstrike [7]	✖	✖	✖	✖	✖	SW	**—**
Tru-D Smart UVC [8]	✖	✖	✔	✖	✔	✖	**—**
UVD Robot [9]	✖	✖	✔	✔	✔	✖	2 h
Helios [10]	✖	✖	✔	✖	✖	✖	**—**
BKS-UVRobot-200 [11]	✖	✖	✔	✔	✔	SW	1.8 h
Honeywell Cabin S. [12]	✖	✖	✔	✖	✖	✖	**—**
Keenon M2 robot [13,14]	✖	✖	✖	✔	✔	✖	**—**
SIFROBOT-6.59 [15]	✖	✖	✖	✔	✔	✖	3 h
ZENZOE [16]	✖	✖	✖	✖	✖	✖	2.7 h
FARYUAN FYB-K3 [17]	✖	✖	✖	✔	✖	✖	6 h

^1^ Live video feedback refers to the possibility of checking the status of the system with the live video feed at any time from anywhere. ^2^ Object recognition refers to the ability of a robot to understand and identify various objects that are inside its visual field. ^3^ Control refers to the integration of functions that enable the control of a robot’s actions from an external source. ^4^ Autonomous mode. ^5^ Upgrade refers to the capacity of a modular system to be openly updated with new software and hardware components. In this table, the term “SW” refers to the possibility of receiving software updates. ^6^ The operation time of the robot while disinfecting is stated in the brochures or website.

**Table 3:** State of the art in the literature of disinfecting robot systems.

Name	Disinfection System	Human Detection System
Ultrabot [18]	Mobile base with 8×vertical static LMP UV-C (4 on each side)	Partially; depth sensing cameras (proximity detection only)
G-Robot [19]	Robot arm (5DOF) wtih 25×LED Far-UVC Lights	NO; Far-UVC light is safer, wavelengths range 207–222 nm
UVC-PURGE [20]	Mobile base with 6×vertical static LMP UV-C	NO
COVID-Bot [21]	Mobile base with 3×vertical static small LMP UV-C	NO
NLA-110 [22]	Static base with 4×vertical static LMP UV-C	NO
D. Robot 1 [23]	Mobile base with 1×static small LMP UV-C	NO; when disinfection starts the doors are locked
D. Robot 2 [24]	Mobile base with 7×vertical and horizontal static LMP UV-C and one UV-C spot placed on the arm	NO

**Table 4:** State of the art in the literature of disinfecting robot systems extra features.

Name	Live F. ^1^	OR ^2^	Ctrl ^3^	Aut. ^4^	Reports	Upgrade ^5^
Ultrabot [18]	✖	✖	✔	✔	✖	✖
G-Robot [19]	✖	✖	✔	✔	✖	✖
UVC-PURGE [20]	✔	✖	✔	✔	✖	✖
COVID-Bot [21]	✖	✖	✖	✔	✖	✖
NLA-110 [22]	✖	✖	✖	✖	✖	✖
D. Robot 1 [23]	✖	✖	✖	✔	✖	✖
D. Robot 2 [24]	✔	✖	✔	✖	✖	✖

^1^ Live video feedback means to check, at any time from anywhere, the status of the system. ^2^ Object recognition refers to the abilities of a robot to understand and identify various objects that are inside its visual field. ^3^ Control refers to the integration of functions that enable the control of a robot’s actions from an external source. ^4^ Autonomous mode. ^5^ Upgrade refers to the capacity of a modular system to be openly updated with new software and hardware components easily by anyone in the domain.

**Table 5:** List of components of the RoboCoV Cleaner robot.

Component	Qty	Component	Qty
PIR Sensor HC-SR501	4	Microchip ATmega2560	1
Ultrasonic Sensor HC-SR04	8	Raspberry Pi 4 Model B—8GB	1
IR Sensor TCRT5000	4	7″ Capacitive Touch LCD 800 × 480	1
12 V Battery Level Sensor	1	Slamtec RPLIDAR A1M8 2D 360	1
QTR-8A Reflectance Sensor	1	DC to DC Converter	1
Pololu 24v18 Motor Driver	1	12 V 10 Ah Battery	1
Pololu DC Motor (100:1) 12 V	2	Pololu 37Dmm Aluminum Standoff	2
Rubber Swivel Caster Wheel	1	360-degree IR Camera	1
4-Channel Relay Module 5V	1	MDF Plate	1
12 V 42 W White 6000K Power LED	1	Nuts, Bolts of Various Sizes	— ^*^
Philips 6 W T8 Tube Ballast	5	Various AWG Wires	— ^*^
Philips 18 W Mercury UVC Lamps	5	Microchip ATMEL-ICE Debugger	1
12V–220 V Inverter	1		

* This omission was due to their classification as consumable items in our analysis.

## Data Availability

Data are contained within the article.

## Abbreviations

The following abbreviations are used in this manuscript:

AAL	Ambient assisted living
AFCO	Graduates in front of companies conference
AMR	Autonomous mobile vehicle
AWG	American wire gauge
AFCO	Graduates in front of companies
BOM	Bill of materials
COVID	Coronavirus disease
DC	Direct current
DNA	Deoxyribonucleic acid
FFP2	Filtering Facepiece Respirator Level 2
HAAR	Haar-like features
IESC	Electrical engineering and computer science
IR	Infrared
LCD	Liquid crystal display
LED	Light emitting diode
LiDAR	Light detection and ranging
LMP	Lower-pressure mercury
MDF	Medium-density fiberboard
MCU	Microcontroller unit
NEIDL	National Emerging Infectious Diseases Laboratories
PIR	Passive infrared
PXF	Pulsed xenon flash
QR	Quick response (code)
RISC	Reduced instruction set computer
RNA	Ribonucleic acid
RGB-D	Red green blue—depth
RPLIDAR	Rotary planar LiDAR
SAATI	Advanced Systems in Automation and Information Technology
SARS-CoV-2	Severe acute respiratory syndrome coronavirus 2
SCSS	Student Scientific Communications Session
SLAM	Simultaneous localization and mapping
SW	Software
UCC	Control central unit
USA	United States of America
US	United States
USSA	Secondary sensor and actuator unit
UV	Ultraviolet light
UV-A	Ultraviolet light type A
UV-B	Ultraviolet light type B
UV-C	Ultraviolet light type C
YOLO	You Only Look Once
